# Crevasse density, orientation and temporal variability at Narsap Sermia, Greenland

**DOI:** 10.1017/jog.2023.3

**Published:** 2023-10

**Authors:** Maximillian Van Wyk de Vries, James M. Lea, David W. Ashmore

**Affiliations:** 1 Department of Geography and Planning, University of Liverpool, Liverpool L69 7ZT, UK; 2 School of Geography and the Environment, University of Oxford, Oxford OX1 3QY, UK

**Keywords:** Crevasses, glacier flow, remote sensing

## Abstract

Mass loss from iceberg calving at marine-terminating glaciers is one of the largest and most poorly constrained contributors to sea-level rise. However, our understanding of the processes controlling ice fracturing and crevasse evolution is incomplete. Here, we use Gabor filter banks to automatically map crevasse density and orientation through time on a ~150 km^2^ terminus region of Narsap Sermia, an outlet glacier of the southwest Greenland ice sheet. We find that Narsap Sermia is dominated by transverse (flow-perpendicular) crevasses near the ice front and longitudinal (flow-aligned) crevasses across its central region. Measured crevasse orientation varies on sub-annual timescales by more than 45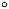 in response to seasonal velocity changes, and also on multi-annual timescales in response to broader dynamic changes and glacier retreat. Our results show a gradual up-glacier propagation of the zone of flow-transverse crevassing coincident with frontal retreat and acceleration occurring in 2020/21, in addition to sub-annual crevasse changes primarily in transition zones between longitudinal to transverse crevasse orientation. This provides new insight into the dynamics of crevassing at large marine-terminating glaciers and a potential approach for the rapid identification of glacier dynamic change from a single pair of satellite images.

## 1. Introduction

The future response of the Greenland and Antarctic ice sheets to changing climatic conditions remains one of the most uncertain factors in forecasts of future sea-level rise (Nowicki and others, 2016; Pörtner and others, 2019; Goelzer and others, 2020; Masson-Delmotte and others, 2021). The majority of the mass loss of these ice sheets occurs at marine-terminating glaciers, which lose mass in part by calving icebergs directly into the ocean (Benn and others, 2007; Vieli and Nick, 2011). Changes in the frontal conditions of marine-terminating glaciers can drive rapid glacier retreat (Venteris, 1999; Nick and others, 2009; Kochtitzky and Copland, 2022), and can promote rapid ice thinning far from the ice front due to dynamic glacier changes (e.g. Pritchard and others, 2009).

Understanding the retreat rates of marine-terminating glaciers relies on identifying the glacier dynamic conditions. Glacier dynamic conditions and changes are currently assessed primarily based on ice velocity calculated using feature tracking, which relies on the existence of coherent satellite image pairs with a suitably low temporal baseline. In the case of marine-terminating glaciers, conditions in fast-flowing regions and close to the calving front often cannot be resolved due to decorrelation or calving ice losses between the two images. The region immediately up-glacier of the ice front is critical for our understanding of calving processes, so in this study, we develop a method for the identification of glacier crevasse patterns from a single image.

Mass loss at marine-terminating glaciers occurs partly through iceberg calving, as fractures propagate through the entire thickness of the glacier. Glacier flow continuously delivers new ice from inland to the glacier front. A glacier's terminus position is stable if the rate of frontal ablation is approximately equal to the flux of ice to the front, and retreating if the rate of frontal ablation exceeds the ice flux. Iceberg calving can drive rapid frontal ablation and glacier retreat (10^2^–10^3^ m a^−1^), particularly where glaciers terminate in deep water (Venteris, 1999; Rivera and others, 2012; Bondzio and others, 2018; Moffat and others, 2018). In order for an iceberg to calve, a fracture must penetrate through the entire ice thickness and separate one or many pieces of ice from the rest of the glacier. These fractures are known as crevasses and are widely present in glacial environments.

**Fig. 1.**
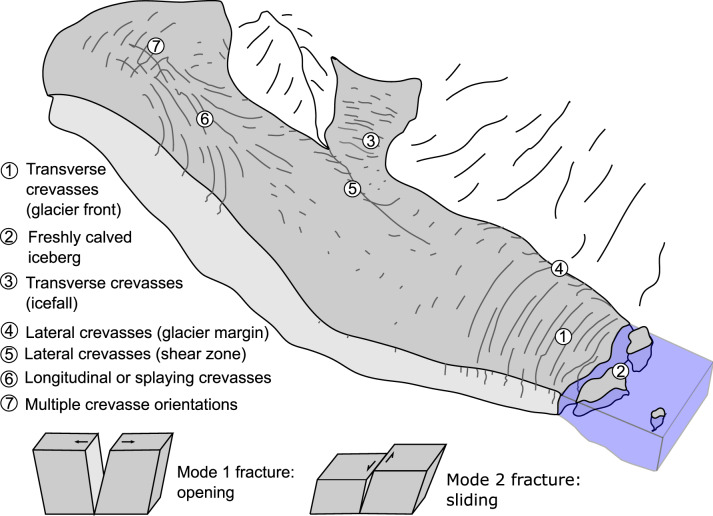
Diagram describing the main categories of crevasses, and their formation mechanisms.

**Fig. 2.**
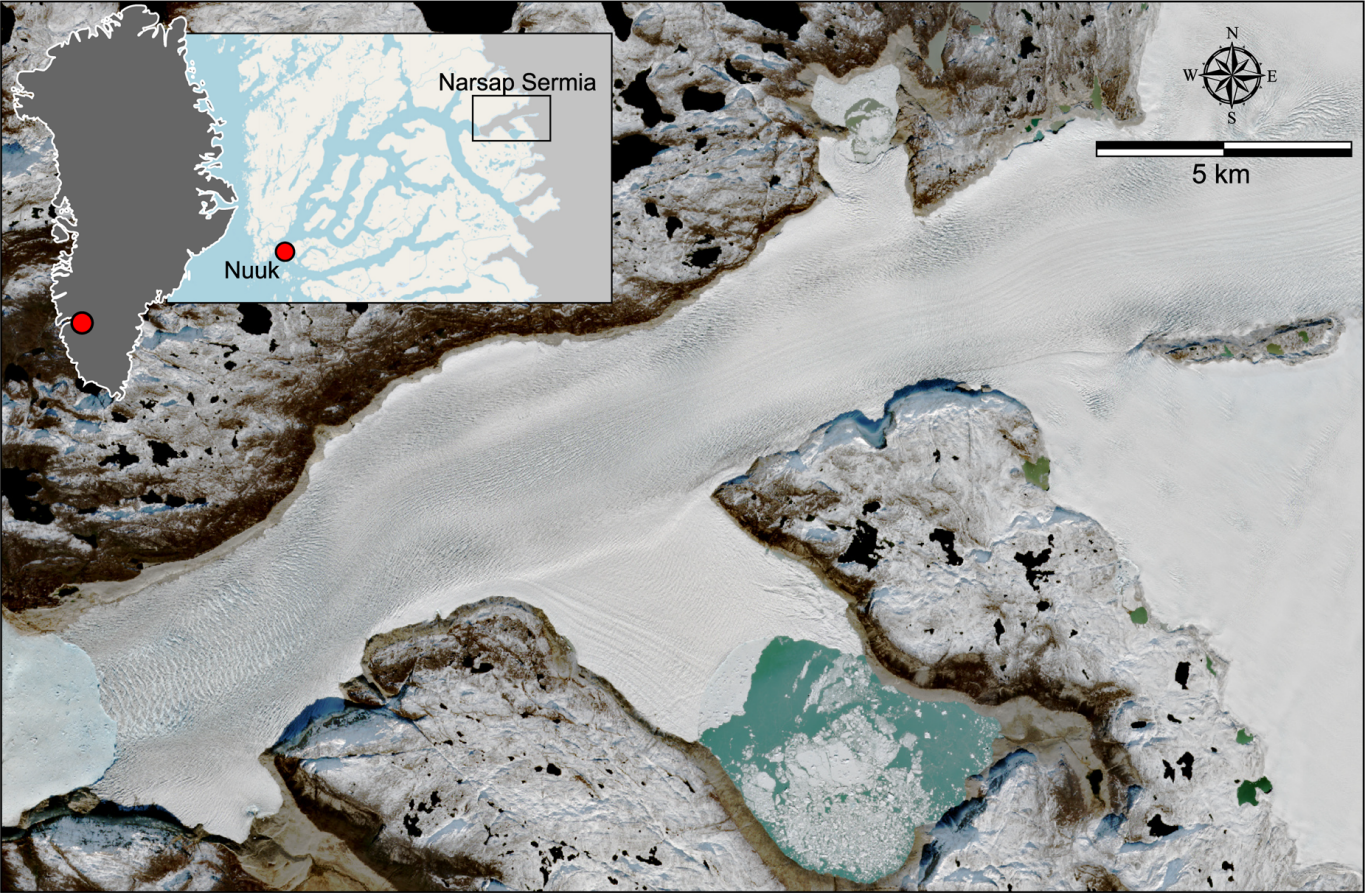
Location of Narsap Sermia in Greenland and within Nuup Kangerlua, and view of the glacier from Sentinel-2.

Cracks form due to tensile or shear stresses in the ice which locally exceed its strength, and may evolve into crevasses (Fig. 1). Crevasses are present on glaciers across a wide range of scales, from metre scale fractures in valley and cirque glaciers to rifts tens of kilometres long at the margins of the Antarctic ice sheet. Crevasses are thought to form approximately perpendicular to the principal normal stress direction: transverse or flow-perpendicular crevasses (oriented ~90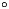 from the direction of ice flow; mode 1) are commonly found in zones of longitudinal extension, while longitudinal or splaying crevasses (oriented approximately parallel to the direction of ice flow; mode 2) are commonly found in zones of longitudinal compression (Fig. 2). Lateral shear at the margins of a glacier often results in crevasses oriented ~45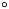 from the valley walls (Nye, 1952; Glen and Perutz, 1955; Nye, 1955; Hudleston, 2015; Colgan and others, 2016; Jennings and Hambrey, 2021). Creep closure usually limits crevasse depth to a few tens of metres, and understanding the conditions under which crevasses may propagate through the entire ice column is key to evaluating the drivers of iceberg calving (Nye and Mott, 1953; Nye and Perutz, 1957; Benn and others, 2007; Mottram and Benn, 2009).

A number of analytical and numerical approaches have been used to evaluate the influence of crevassing on glacier calving, from simple ‘zero-dimensional’ calving laws to full 3-D glacier models (e.g. Nye and Perutz, 1957; van der Veen, 1998; Pralong and Funk, 2005; Mottram and Benn, 2009; Krug and others, 2014; Choi and others, 2018; Todd and others, 2018; Bassis and others, 2021; Crawford and others, 2021; Cook and others, 2022). Certain early studies of calving bypass crevasses entirely, and relate glacier calving rate to the water depth at the ice front (Brown and others, 1982; van der Veen, 1998) or ice front height (Pfeffer and others, 1997). These empirical formulations allow models to reproduce observed rates of ice retreat in some cases but are not physically based. More recent studies have proposed different calving models related to ice dynamics and physical variables, for instance, height-above-buoyancy models (Vieli and others, 2001), crevasse depth criteria (Benn and others, 2007), von Mises tensile stress criteria (Morlighem and others, 2016; Choi and others, 2018), kinematic calving laws (Levermann and others, 2012) and linear elastic fracture mechanics approaches (Yu and others, 2017). Crevasses are commonly parameterized in numerical models as damage fields, accounting for the bulk effect of brittle fractures on viscous ice flow (Pralong and others, 2003; Pralong and Funk, 2005; Borstad and others, 2012; Lhermitte and others, 2020). Advances in computational power have also enabled the use of discrete element models to simulate crevasse calving, which can reproduce individual calving events in greater detail (Bassis and others, 2017; Cook and others, 2018; Gong and others, 2018; van Dongen and others, 2020). Many advances in the numerical description of crevasses have been made over the past 20 years, but limitations remain in our understanding and numerical representation of crevasse penetration at the ice front, and ice-sheet models lack a universally applicable and computationally efficient representation of calving. Large-scale observations of crevasse evolution are therefore key to developing and evaluating new crevasse models.

Crevasses form visible traces on the surface of glaciers, and can therefore be identified and monitored remotely via ground, air or satellite-based imagery. Manual identification of crevasses in remotely sensed imagery dates back to almost 50 years (e.g. Krimmel and Meier, 1975), and has been reviewed by Colgan and others (2016) and Jennings and Hambrey (2021). The study of glacier surface crevasse patterns is a key method in structural glaciology and can yield insight into glacier strain history, current dynamics and future stability. Structural glaciology is a sub-discipline that aims to evaluate glacier processes using their structural characteristics: crevasses, faults, folds, foliation and more (Hudleston, 2015; Jennings and Hambrey, 2021). For instance, crevasses were manually mapped at Worthington Glacier, USA (Harper and others, 1998), Haut Glacier d'Arolla, Switzerland (Goodsell and others, 2005), Kvíárjökull, Svínafellsjökull and Fjallsjökull, Iceland (Phillips and others, 2017; Swift and others, 2018; Dell and others, 2019, respectively), Isunguata Sermia, Greenland (Jones and others, 2018) and Fountain Glacier, Canada (Jennings and others, 2022).

Semi-automated crevasse mapping methods are also used, including the use of histogram equalization or edge enhancement image processing to facilitate manual crevasse identification (Colgan and others, 2011, 2016). However, manual or semi-automated crevasse identification is both subjective and time-consuming, preventing multi-temporal mapping of crevasses over large spatial areas. Therefore, a range of fully automated crevasse mapping techniques have also been developed: Bhardwaj and others (2016) used a simple Landsat band ratio technique to map small crevasses on Shaune Garang glacier (Himalaya), Gong and others (2018) used the Radon transform to evaluate linear features on a surging outlet of Austfonna (Svalbard), Chudley and others (2021) compared a Random Forest classification to crevasse maps from high-resolution elevation data in Greenland, Christmann and others (2021) compared DEM-derived and manually mapped crevasse fields over Greenland to stress change maps, Lai and others (2020) used a neural network to identify Antarctica-wide rifts using MODIS Mosaic of Antarctica imagery, Zhao and others (2022) applied deep learning to Sentinel-1 radar imagery to map fractures in Antarctic ice shelves, and Izeboud and Lhermitte (2023) develop a method for automated crevasse detection using a normalized radon transform. Despite the recent advances in automated crevasse mapping, most existing techniques either require an extensive training dataset (e.g. machine learning-based methods; Lai and others, 2020; Chudley and others, 2021; Zhao and others, 2022), or only provide overview statistics instead of the location and attributes of individual crevasses (e.g. Radon transform-based methods; Gong and others, 2018).

Here, we describe a new automated crevasse mapping method based on processing images with linear feature detection kernels: Gabor filters. This method is computationally fast, does not require any training data and provides a map of individual crevasse locations. We apply this method to automatically map the occurrence of crevasses on 6 years of Sentinel-2 data (224 images) on a ~150 km^2^ region of Narsap Sermia, an outlet glacier of the Greenlandice sheet. We also calculate glacier velocity maps so as to compare crevasse fields to glacier velocity magnitude and direction. We use these data to explore the following three research questions:

1. What is the spatial pattern and orientation of crevasses across a marine-terminating glacier?
2. Is the distribution, extent and orientation of crevasses constant through time, or does it exhibit seasonal and/or multiannual changes?
3. What is the relation between crevasse characteristics and glacier velocity?

## 2. Study area

Narsap Sermia is an outlet glacier draining the southwest margin of the Greenland ice sheet, and the closest marine-terminating glacier to Greenland's capital and most populous city, Nuuk (Pearce and others, 2018). Alongside Kangiata Nunaata Sermia and Akullerssuup Sermia, Narsap Sermia calves icebergs directly into Nuup Kangerlua (Nuuk Fjord), which can be advected down-fjord and have the potential to impede shipping routes to Nuuk. The frontal position of Narsap Sermia remained close to its Little Ice Age position until the early 21st century (Motyka and others, 2017), whereupon it has exhibited several episodes of rapid ice retreat (Motyka and others, 2017; Davison and others, 2020), the latest of which is ongoing today. Narsap Sermia currently terminates in moderate to shallow depth water (~200 m; Morlighem and others, 2014), but a deep subglacial trough is present ~5–20 km up-glacier from the 2022 calving front (Morlighem and others, 2014; Motyka and others, 2017). If Narsap Sermia's calving front were to reach the retrograde slope at the down-glacier edge of this subglacial trough, it could enter a phase of runaway retreat observed at other marine-terminating glaciers (Venteris, 1999; Motyka and others, 2017). This runaway retreat might also be associated with changes in calving style, affecting the quantity and size distribution of icebergs advected towards Nuuk. Narsap Sermia has three subsidiary ice fronts terminating in ice-marginal lakes, including the ~20 km^2^ glacial lake Iluliartôq. All three lakes have varying volumes and drain under the glacier, with the lowermost lake remaining fully drained since the latest episode of frontal retreat (post-2014). The societal importance of calving processes at Narsap Sermia, along with its high degree of crevassing and ongoing frontal retreat, make it an ideal case study for spatiotemporal changes in crevassing.

## 3. Methods

**Fig. 3.**
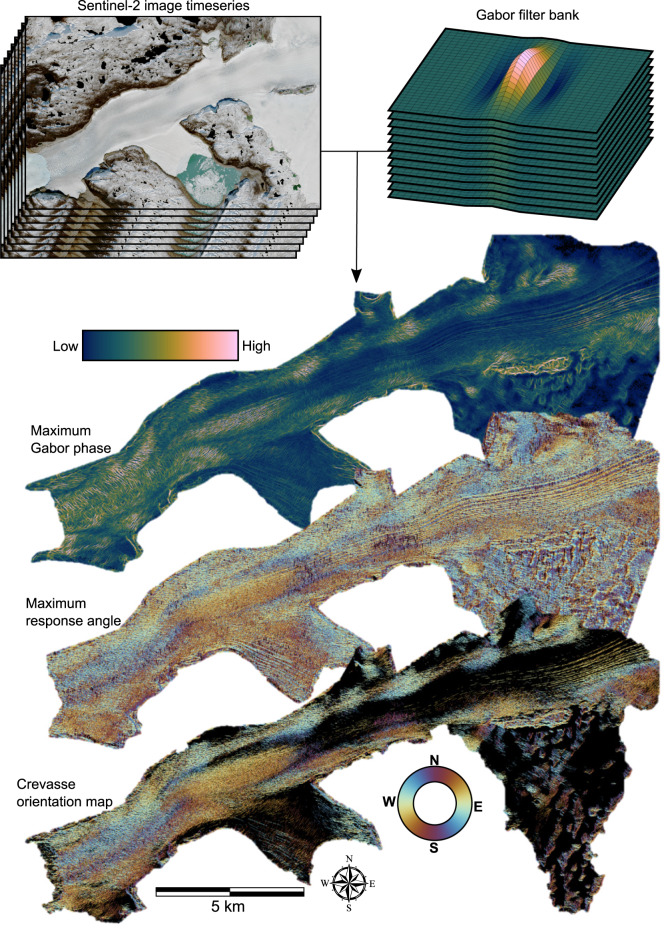
Diagram of the main steps involved in the automated crevasse mapping method. A time series of satellite imagery is convolved with a Gabor filter bank, creating maximum Gabor phase and maximum response angle maps. The maximum Gabor phase map is then thresholded to extract a crevasse network, with the orientation of each crevasse obtained from the maximum response angle map.

We implement a method to automatically detect and calculate the orientation of crevasses within satellite images using a Gabor filter bank. A Gabor filter is composed of a sinusoidally modulated Gaussian kernel and is sensitive to image texture. By combining a bank of filters of different orientations, we extract individual crevasse locations and orientations from optical satellite images (Fig. 3). We describe the attributes of the Gabor filter bank and crevasse detection in more detail in Appendix A1. This method is computationally rapid, extracts individual crevasse orientations and does not require a training dataset. We apply this method, which we term the Gabor crevasse detector (GCD), to all 10 m resolution near-infrared (band 8) Sentinel-2 L1C images of Narsap Sermia from its operational start in June 2015 to April 2022. We select the near-infrared band as it shows a strong contrast between crevassed and non-crevassed areas, but do not expect major differences if computed with other visible spectrum bands or band combinations. We exclude all images in which the glacier is obscured by cloud cover or shadows, retaining the 224 images in which the entire glacier area of interest is unobstructed. As well as calculating a binary crevasse mask and crevasse orientation map for each image, we calculate three crevasse statistics in 20 × 20 pixel sub-regions. The three statistics are crevasse spatial density (the ratio of pixels classified as crevasses to the total number of pixels in the sub-region), the median crevasse orientation and the mean absolute deviation of the measured crevasse orientation. The mean absolute deviation provides an estimate of the local degree of dispersion of measured crevasse orientations. We evaluate the GCD in Appendix A3 by comparing it to two other, previously used crevasse mapping techniques: digital elevation model (DEM) thresholding and manual delineation.

We use feature-tracking toolbox GIV to calculate 80 m-resolution glacier-surface-velocity and strain maps for Narsap Sermia, using a frequency-domain multi-pass image correlator (Van Wyk de Vries and Wickert, 2021). We use velocity maps to assess glacier surface strain rates, and the orientation of crevasses relative to ice flow direction. We also calculate apparent displacements over the surrounding bedrock to correct for georeferencing errors and evaluate local noise levels. We compile three different datasets of image pairs based on the temporal separation between images: short period (1–8 d), medium period (9–90 d) and long period (300–430 and 650–800 d). This separation allows us to monitor displacements over both regions of fast ice flow in which surface decorrelation occurs rapidly and regions of slow ice flow in which long temporal separation is necessary for resolvable displacement to occur.

We calculate velocities from a total of 7194 image pairs, of which 330 are short-period image pairs, 2306 are medium-period image pairs and 4558 are long-period image pairs. Calculating a large number of individual velocity maps improves the precision of median velocity maps (Millan and others, 2019; Van Wyk de Vries and Wickert, 2021). A full description of our velocity mapping workflow is provided in Appendix A2. We average all processed velocity maps into a median velocity map covering the entire 6 year period. We also calculate annual median velocity maps for each of the three image temporal separations (short, medium and long). We calculate longitudinal (*σ*_*xx*_), transverse (*σ*_*yy*_) and shear (*σ*_*xy*_) strain rate maps from each of the overall and annual median velocity maps. Finally, we calculate the relative angle between measured crevasse orientation (calculated using the GCD described above) and local ice-flow direction and classify crevasses into three categories: longitudinal crevasses oriented close to (0–33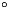) of the direction of ice flow, transverse crevasses oriented approximately perpendicular to the direction of ice flow (66–90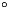) and crevasses oriented in neither of these directions (33–66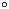).

## 4. Results

**Fig. 4.**
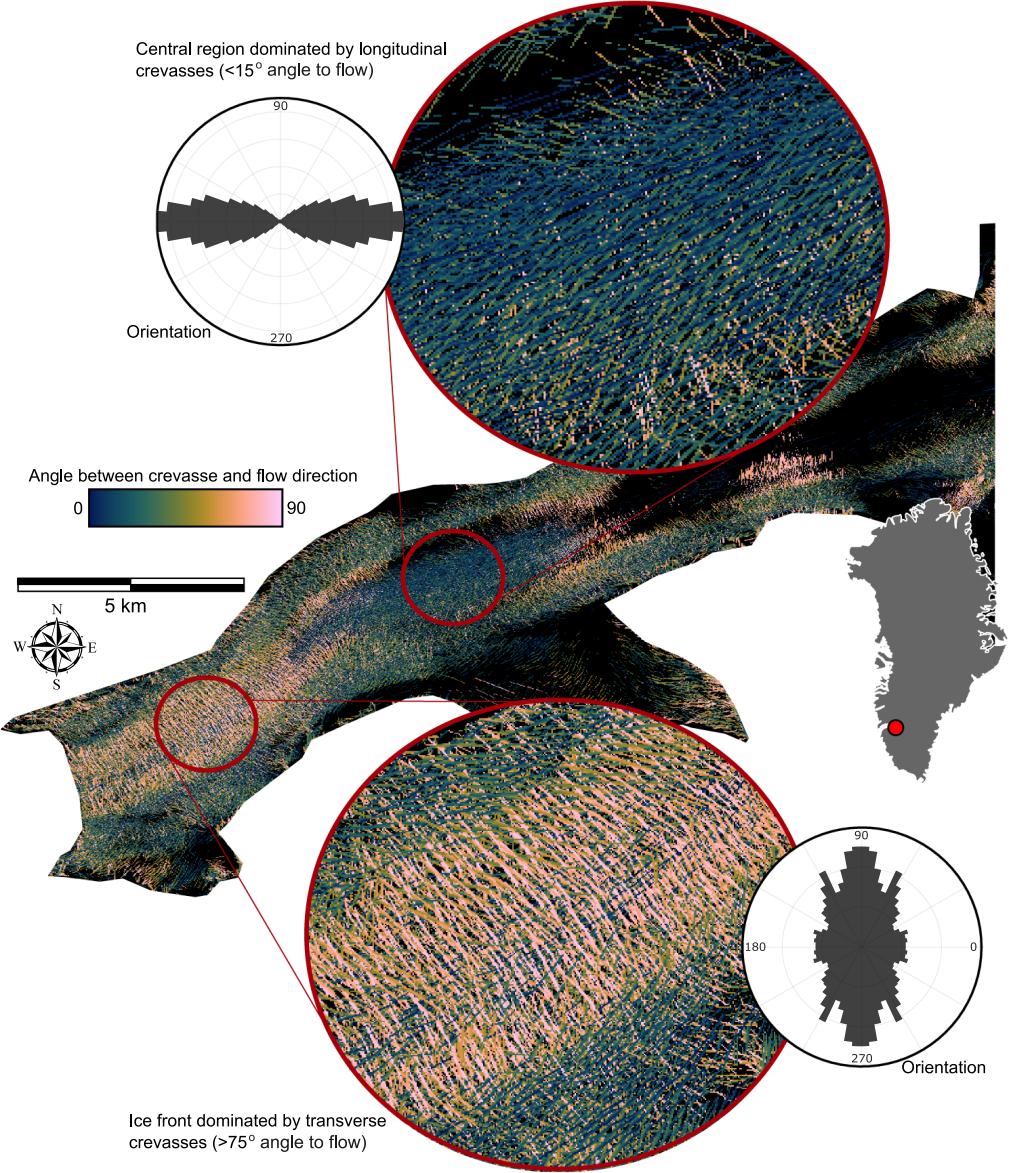
Measured crevasse orientation map of Narsap Sermia from 17 April 2022, with inset showing the two key crevasse zones: a zone of transverse crevassing near the ice front, and a zone of longitudinal crevassing up-glacier of this. The polar histograms show the angle between the direction of crevassing and the direction of ice flow in each inset.

We find an overall decrease in measured crevasse density with distance away from the calving front at Narsap Sermia, with three main styles of crevassing (Fig. 4). The first type of crevasse, oriented approximately perpendicular to the direction of ice flow, is abundant within 5 km of the glacier marine calving front and to a lesser degree at subsidiary lacustrine calving fronts. These crevasses form large, arcuate fractures in the extensively damaged zone proximal to the calving front. The second type of crevasses, oriented ~45 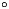 from the direction of ice flow, are most abundant on the flanks of the zone of fast ice flow at the centre of the valley. The third type, oriented approximately parallel to the direction of ice flow, is primarily located within the centre of the glacier valley. A 3 km long by 1 km wide region located halfway up the outlet glacier (~10 km from the ice front) is dominated by this third, longitudinal crevasse type. While the first, transverse, crevasse type is the most abundant close to the ice front, the second and third crevasse types are also present to a lesser degree.

**Fig. 5.**
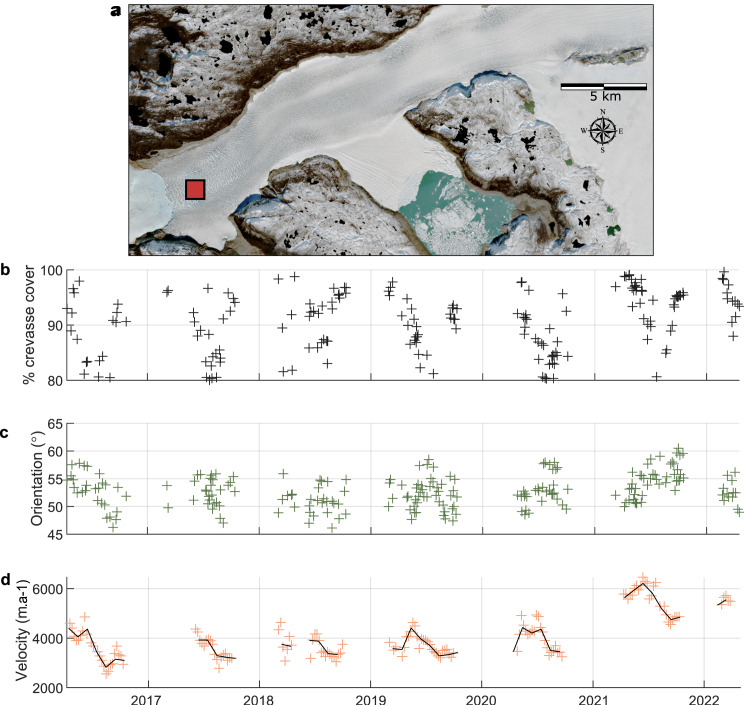
Time series of measured crevasse density (b), measured crevasse orientation relative to the direction of ice flow (c) and ice velocity (d) for the 1 km^2^ down-glacier zone at the front of Narsap Sermia (a). The dark line represents the median monthly velocity. Note that the *y*-axes are different to Figs 6 and 7 to highlight the seasonal variation in each location.

**Fig. 6.**
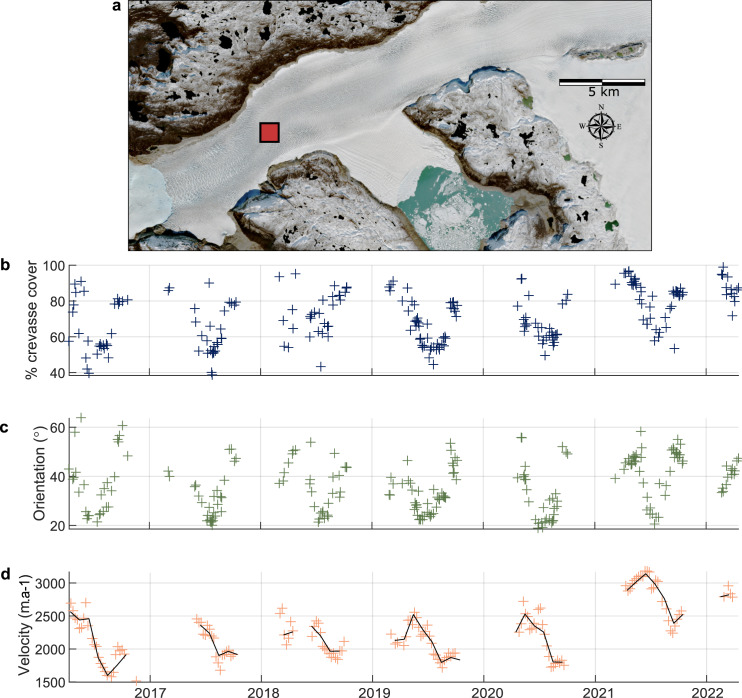
Time series of measured crevasse density (b), measured crevasse orientation relative to the direction of ice flow (c) and ice velocity (d) for the 1 km^2^ transitional zone ~5 km up-glacier from the front of Narsap Sermia (a). The dark line represents the median monthly velocity. Note that the *y*-axes are different to Figs 5 and 7 to highlight the seasonal variation in each location.

**Fig. 7.**
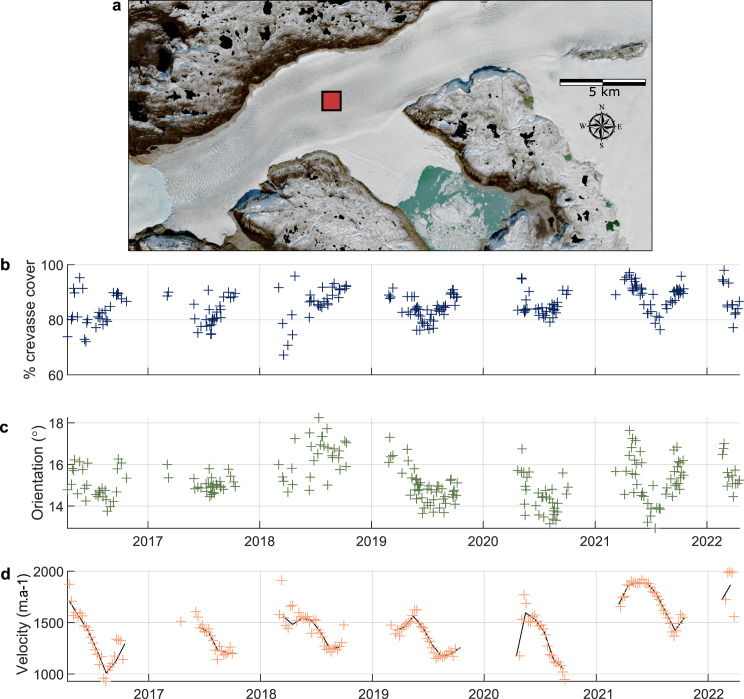
Time series of measured crevasse density (b), measured crevasse orientation relative to the direction of ice flow (c) and ice velocity (d) for the 1 km^2^ up-glacier zone ~10 km up-glacier from the front of Narsap Sermia (a). The dark line represents the median monthly velocity. Note that the *y*-axes are different from Figs 5 and 6 to highlight the seasonal variation in each location.

**Fig. 8.**
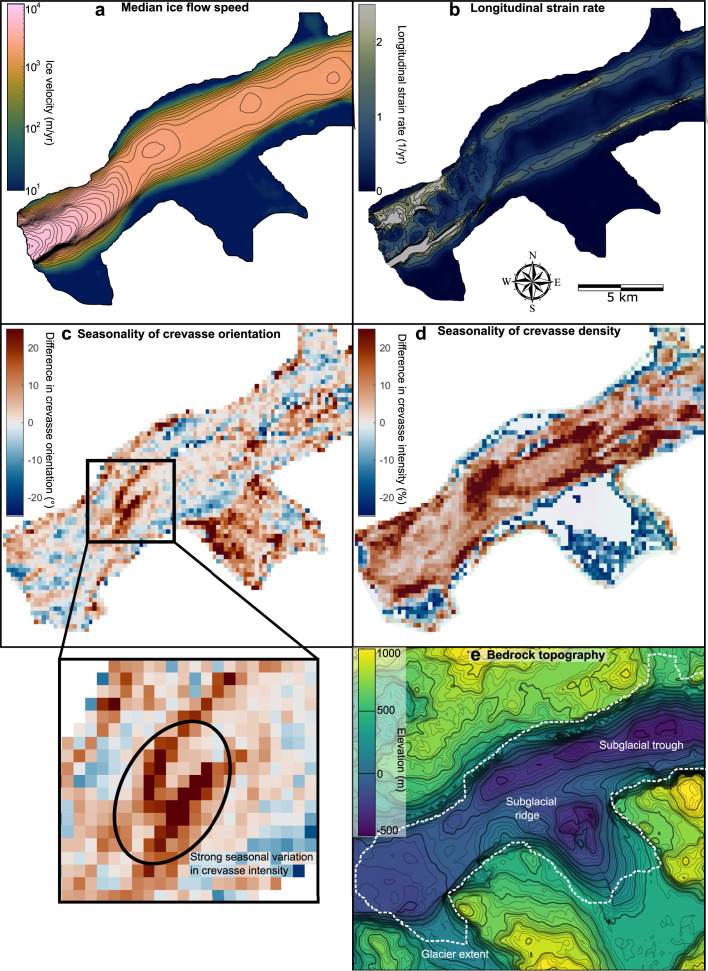
Median ice flow speed (a), effective strain rate (b), measured crevasse orientation seasonality (c), crevasse density seasonality (d) and subglacial topography (e). Measured crevasse orientation and density seasonality are calculated as the average of September 2020 to May 2021 minus the average of June 2020 to July 2020. Note that the apparently large seasonal changes on the southern lake-terminating glacier are highly uncertain, and we do not interpret these. Subglacial topography is obtained from BedMachine v4.0 and is referenced to the local mean sea level (geoid).

We assess crevasse and velocity changes at three 1 km^2^ zones on the glacier representing these main regions: the first ‘down-glacier’ zone is located close to the ice front in the zone of longitudinal crevassing (Fig. 5), the second ‘transitional’ zone is located 7.5 km up-glacier at the transition between the longitudinal crevassing and transverse crevassing zones (Fig. 6) and the third ‘up-glacier’ zone is located within the zone of transverse crevassing (Fig. 7). The down-glacier zone is located in a region of moderate depth-to-bedrock (~250 m ice thickness; BedMachine v4.0), low slope subglacial topography while the transitional zone is on the downstream tip of a subglacial ridge and the up-glacier zone is at the centre of the same subglacial ridge and close to its transition into a subglacial trough (Fig. 8e).

Crevasses also exhibit seasonal variability, with strong seasonal changes in both the intensity and dominant orientation of crevasses in some regions of Narsap Sermia. Measured crevasse density remains high throughout the year in the down-glacier zone, with close to 100% crevasse coverage throughout the year (Fig. 5a). The intensity of secondary (45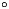 and longitudinal) crevassing exhibits minor variability, although transverse crevassing remains dominant at all times. The median angle between measured crevasse orientation and ice flow in the down-glacier zone varies between 55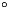 and 75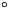 (Fig. 5b). Seasonal variability in both measured crevasse density and orientation is high at the transitional zone, with a summer minimum in the degree of crevassing (Fig. 6a). Measured crevasse density reaches close to 100% in the winter and spring, but declines to <50% in the mid-summer (Fig. 6b). The degree of summer (July) crevassing in the transitional zone has increased from 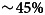 in 2016 to 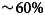 in 2021. The median angle between measured crevasse orientation and ice flow in this zone fluctuates from ~20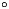 in the summer to 70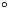 in late autumn to spring (Fig. 6b). In the up-glacier zone the median angle between measured crevasse orientation and ice flow remains low (10–20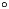) and the intensity of crevassing remains close to 100% throughout the year (Figs 7a, b).

Ice velocities show a similar trend in all three of these zones, with a seasonal minima in the late summer and maxima in the early spring (Figs 5c, 6c, 7c). Velocities also show a long-term acceleration, with 2021 velocities being ~33% higher than in 2016. In the transitional zone, the yearly velocity maxima coincide with the yearly minima in measured crevasse density and angle between measured crevasse orientation and ice flow (Fig. 6).

The region of high seasonal variation in measured crevasse orientation is spatially limited to the boundary between the frontal region of transverse crevasses and the up-glacier zone of longitudinal crevasses. This region exhibits a 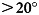 variation in median angle between measured crevasse orientation and ice flow throughout the year (Fig. 8c). A broader region exhibits a moderate to high seasonal variation in measured crevasse density, with the surroundings of the up-glacier zone of longitudinal crevasses all exhibiting a ~20% seasonal variation in measured crevasse density (Fig. 8d). The up-glacier zone of longitudinal crevassing is located above a subglacial ridge (Fig. 8e) and in an area with very low velocity gradients and effective strain rates (Figs 8a, b).

## 5. Discussion

### 5.1 Key findings and limitations

Our automatically processed crevasse orientation maps, based on 6 years of Sentinel-2 data and 224 individual images of Narsap Sermia, provide the first dense time series of changes crevasse change at a major glacier. We first show that the large-scale pattern of crevassing remains consistent across the entire study period, with a zone of longitudinal crevassing near the glacier front and a zone of transverse crevassing up-glacier of this. These persistent large-scale crevasse patterns likely relate to the glacier geometry and bed elevation. Conversely, we identify both strong seasonal and multiannual variability when examining the time series of measured crevasse density (the spatial density of crevasses in a given area) and orientation (the median difference between crevasse angle and ice-flow direction). The main glacier trunk exhibits a seasonal minimum in the degree of crevassing in the late summer, and some regions exhibit a seasonal fluctuation between transverse crevassing (high angle to ice-flow direction) to longitudinal crevassing (low angle to ice-flow direction). Velocity mapping highlights a multiannual increase in ice flow speeds with the greatest increase close to the ice front (Figs 5d, 6d, 7d), which is a possible cause for observed multi-annual increases in measured crevasse density and changes in measured crevasse orientation 5–10 km up-glacier from the ice front.

Our GCD crevasse mapping method can be leveraged to rapidly map the characteristics of thousands of individual crevasses or whole crevasse fields. Using automated instead of manual crevasse mapping methods can enable structural glaciology techniques to be upscaled from single glaciers and time periods to whole ice caps and/or multiple time periods. Our results from Narsap Sermia show that a crevasse map derived from a single time period might not be representative of the glacier's overall conditions. The first implication of this finding is that glacier structure may be more dynamic than previously considered (Hudleston, 2015; Jennings and Hambrey, 2021), and be a valuable tool for assessing short-timescale glacier change. The second implication is methodological: studies aiming to use crevasses to map a glacier's dynamic conditions must account for this temporal variability, as manually mapping crevasses over a single time step may produce a map reflecting transient conditions at this point in time, rather than the longer timescale forcings of interest (e.g. Phillips and others, 2017; Jones and others, 2018; Swift and others, 2018; Dell and others, 2019; Jennings and others, 2022).

While the GCD described in this study has a number of advantages, it also has certain limitations. An ideal crevasse model would be able to delineate all crevasses in a glacier, regardless of their scale or physical appearance, while also not falsely classifying any other glacier surface features as crevasses. The complex and highly variable nature of a glacier surface makes this a complex task: glaciers can exhibit a wide range of surface expressions (e.g. Herzfeld and others, 2004; Colgan and others, 2016) and features other than crevasses can form linear expressions on a glacier surface, including medial moraines, longitudinal foliation, ogives and flow banding (Hudleston, 2015; Jones and others, 2018; Jennings and Hambrey, 2021). The GCD is in most cases unable to distinguish between linear expressions on the ice surface related to crevassing and those related to other processes. The GCD can identify sub-pixel resolution crevasses (<10 m in the case of Sentinel-2), but requires them to leave distinct surface expressions, and will likely fail where multiple small crevasses are present within a single pixel. The GCD only identifies a single-dominant crevasse orientation at any given pixel, which can result in information loss at pixels where two or more crevasses of different orientations intersect. Finally, the GCD requires a surface expression of crevasses in optical imagery, and will fail to identify crevasses entirely buried by snow (which can be identified using other methods; e.g. Eder and others, 2008; Thompson and others, 2020). The use of SAR data could enable crevasse detection in cases where it is not possible in optical data, but we limit our use of the GCD to optical data in this study. Crevasse surface expression and degree of shadowing can also be affected by sun azimuth. We, therefore, suggest that the GCD be primarily used in areas where (1) crevasses are close to or greater than the resolution of the imagery used to map them, (2) crevasses are the dominant structural feature on the glacier surface and (3) the glacier surface is largely free of snow cover.

### 5.2 Drivers of seasonal cycle

The seasonal variation in crevasse characteristics observed (density and orientation) could be an artefact of other seasonal changes, such as a seasonal change in snow cover or illumination. We critically evaluate the possibility of each of these in this section and discuss why we think the observed seasonal variation on the main branch of Narsap Sermia represents a real signal. **Illumination:** At a latitude of more than 64° N, Narsap Sermia is subject to a strong seasonal cycle in illumination. This could plausibly cause an apparent seasonal cycle in crevassing by reducing the winter illumination and modifying illumination angles throughout the year. We plot the seasonal change in midday sun azimuth (variation between 198° and 213°) and midday sun elevation (variation between 2° and 46°) alongside the seasonal change in crevassing in the transitional zone in Supplementary Section S2, with the two being anti-correlated. However, with the exception of shadowed areas, we would expect the effect of illumination to be approximately constant across the glacier for crevasses of a given orientation. Our seasonal variability results are not consistent with a change in illumination alone, as both the seasonality in measured crevasse density and orientation are spatially variable across the glacier (Fig. 8). The seasonal variation in crevasse cover and orientation is much higher in the transitional zone than in the up-glacier or down-glacier zones (Figs 5–7). In addition, we manually excluded all winter imagery in which the surface of the glacier is covered by shadows or poorly illuminated, so direct shadowing cannot be a contributor to our observed seasonality.

**Snow cover:** With winter temperatures below freezing and summer temperatures above freezing, Narsap Sermia experiences a seasonal cycle in snow cover. Snow can cover the glacier surface and mask crevasses, or even preferentially cover crevasses of a certain orientation due to a prevailing wind direction (katabatic winds) or differing crevasse sizes. Snow may also cause an apparent spatial gradient in this seasonal variation, with more snowfall and masking of crevasses for a longer duration at higher altitudes. However, we find it unlikely that snow could explain the apparent pattern of crevasse changes as: (1) at Narsap Sermia, crevasses are large enough to never be fully snow-covered. Crevasses are fully visible even in images from mid-winter. (2) We would expect snow to reduce the apparent crevasse density in the accumulation season and reduce it in the ablation season, but the area with the highest seasonal variation in measured crevasse density reaches a minimum during the ablation season (Fig. 6). (3) We expect the effect of snow cover to be either uniform across the glacier or elevation dependant, whereas the observed pattern of seasonal changes is more complex.

Snow cover may be a factor in the marginal areas of Narsap Sermia (e.g. southern lake-terminating branch), which exhibit much slower flow velocities, smaller crevasses and have an apparent summer maximum in crevassing. However, we consider it unlikely that changes in snow cover are a major influence of the observed seasonal cycle in crevassing along the main branch of Narsap Sermia.

We do not consider it likely that artefacts related to seasonal changes in illumination or snow cover are the primary cause of the observed seasonal cycle in measured crevasse density or orientation along the main branch of Narsap Sermia. We also note that the magnitude of this seasonal change is small across most of the glacier (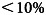 at points 1 and 3; Figs 5, 7) and would likely not be apparent from visual interpretation of satellite imagery alone. Seasonal changes in measured crevasse orientation and density are large in the vicinity of point 2, which is located at the transition zone between longitudinal and transverse crevassing. We show a year's worth of imagery at this point in Supplementary Section S1, which clearly shows that the glacier surface is not shadowed or snow-covered throughout the period.

We hypothesize that this cycle is caused by a seasonal shift in the position at which the transverse crevasses begin to open, with them opening further up-glacier in the glacier in the winter (high measured crevasse density, high crevasse angle to flow direction) than in the summer (lower measured crevasse density, low measured crevasse orientation). This mechanism for seasonal variation in crevassing does not require the closing of crevasses over the course of a season: from the Lagrangian reference frame (advected with ice flow), individual crevasses simply open at different times throughout the year. In the Eulerian reference frame (fixed geographic location) this difference can manifest as a seasonal cycle in crevasse density and orientation as different populations of crevasses are advected past the point. This cycle is consistent with the ice velocity trends, with a summer slowdown apparent across the glacier front (Figs 5, 6, 7d). In the absence of dense time series of crevasse density or orientation at other glaciers, it remains unknown whether such zones of high seasonal variability are widespread at tidewater glaciers.

### 5.3 Crevasses and terminus stability

Our results are not consistent with a binary classification of crevasses into transverse crevasses forming in longitudinal tension, and longitudinal or splaying crevasses forming under longitudinal compression (Glen and Perutz, 1955; Colgan and others, 2016; Jennings and Hambrey, 2021). While we do identify sectors where crevasses are primarily aligned perpendicular or parallel to the direction of ice flow, they retain a wide distribution of orientations (Fig. 4). For example in the proximal region to the ice front dominated by longitudinal crevasses, crevasses are found oriented in all directions: while crevasses oriented perpendicular to the direction of ice flow is the most abundant, there are secondary populations oriented 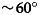 and parallel to the direction of ice flow (Fig. 4). Crevasses are oriented almost exclusively parallel to the direction of glacier flow in the zone 5–10 km from the calving front, yet the angle between crevasses and the direction of ice flow is commonly as high as 20–30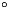. Our results are in line with prior studies which have highlighted the complex relation between velocity, strain and measured crevasse orientation, particularly in the case of crevasse fields with multiple closely spaced crevasses (Sassolas and others, 1996; Harper and others, 1998; Herzfeld and others, 2004; Colgan and others, 2016). This diversity in measured crevasse orientation and its potential impact on calving behaviour is largely unaccounted for in the current generation of ice-sheet models.

In order for calving to occur, a fracture must propagate through the entire thickness of the glacier and separate one or more fragments of the glacier from the main ice mass. Crevasse orientation is, therefore, a first-order control on calving as transverse crevasses are more easily able to separate icebergs than longitudinal crevasses. In an endmember case with crevasses oriented parallel to ice flow, fracture opening will simply cause the ice front to splay but be unable to detach any icebergs. As our results show, this endmember is not realistic as ice-front conditions and lateral shear margins result in a wider range of crevasse orientations, but some glacier fronts do exhibit a distinctive splaying crevasse pattern (e.g. Glaciar San Quintín, Chile; Svïnafellsjökull, Iceland or Sefstrøms Gletscher, Greenland; Paterson, 1961; Swift and others, 2018; Minowa and others, 2021). Crevasse orientation can therefore be both a result of a stable glacier front configuration (for instance with longitudinal compression at a pining point) and itself a contributor to stability (with a high angle between crevasses and the ice front inhibiting rapid iceberg calving). Many of the current generation of calving models are calibrated using 1-D or 2-D cross-sectional glacier models (e.g. Benn and others, 2007; Nick and others, 2010; Bassis and Jacobs, 2013; Krug and others, 2014; Lea and others, 2014; Bassis and others, 2021). These models are inherently unable to capture the impact of crevasse orientation on calving rate. Models using a von Mises calving law (e.g. Choi and others, 2018) incorporate both principal stress directions to scale the calving rate, but this remains a necessarily simplified representation of the true crevasse pattern for inclusion in numerical modelling. Crevasse orientation can be evaluated in 3*D discrete element models (Bassis and others, 2017; Cook and others, 2018; van Dongen and others, 2020), however, these are computationally intensive and cannot easily be coupled to continuum ice-sheet models. Future studies should aim to parameterize the effect of crevasse orientation on calving rate and iceberg size distributions so that structural glaciology observations can be used to improve forecasts of future ice mass loss. High-resolution, spatially extensive and transient crevasse maps as presented in this study may be used to calibrate such models.

### 5.4 Future stability of Narsap Sermia

Finally, we consider the implications of our results for the stability and future evolution of Narsap Sermia. The recent retreat of Narsap Sermia has been well documented in previous studies (Motyka and others, 2017; Davison and others, 2020), and has persisted through our study period. As of early 2022, the front of Narsap Sermia is 1–2 km up-glacier of its 2016 terminus. Can we gain any insight from our crevasse mapping about whether or not Narsap Sermia is likely to retreat into the overdeepened glacial trough? Firstly, we observe a persistent pattern of longitudinal crevasses ~5–10 km up-glacier of the current ice front. This crevasse pattern is diagnostic of compressional longitudinal stresses (Colgan and others, 2016; Jennings and Hambrey, 2021), for instance at a pinning point, and conducive to glacier stability. Compressional ice flow as ice exits the overdeepened trough might inhibit rapid ice retreat. Secondly, we observe an up-glacier migration of the frontal zone of transverse crevassing, with a gradual increase in the angle between crevasses and ice-flow direction at the transition between the transverse and longitudinal crevassing zones (Fig. 6c). We also observe an increase in ice velocity along the entire central lobe of the glacier, with the largest acceleration occurring close to the ice front. This will be associated with increased longitudinal strain and stress. If longitudinal tension increases sufficiently, it might overcome the present-day compressional flow out of the overdeepened trough, increase ice damage and promote rapid calving. If this does occur, it will likely be preceded by continued up-glacier migration of the zone of transverse crevassing, and overprinting of the current patch of longitudinal crevasses. Real-time monitoring of the crevasse conditions at Narsap Sermia can enable rapid identification of any dynamic change preceding rapid retreat.

## 6. Conclusions

We use a novel crevasse detection method to map the crevasse pattern of an entire Greenland outlet glacier, Narsap Sermia, across more than 6 years of Sentinel-2 data. Our crevasse detection method, based on filtering optical satellite images using a Gabor filter bank, is able to classify both the location and orientation of individual crevasses. We use this to identify both spatial and temporal patterns in crevassing. The 5 km of Narsap Sermia closest to the calving front is dominated by transverse crevassing, while the central region of the outlet glacier is dominated by longitudinal crevassing, approximately parallel to the direction of glacier flow. Both measured crevasse density and orientation exhibit strong seasonal variability across much of Narsap Sermia, with a seasonal minimum in crevassing in late spring to early summer coincident with a maximum in ice flow speed. Crevassing and velocity also exhibit multiannual changes, with up-glacier propagation of the zone of transverse crevassing coincident with frontal retreat and acceleration. High-resolution, multi-temporal crevasse maps contribute to our understanding of crevasse and calving dynamics, which facilitates forecasting of glacier dynamic change and remains challenging to represent in numerical glacier models.

## Figures and Tables

**Fig. 9.**
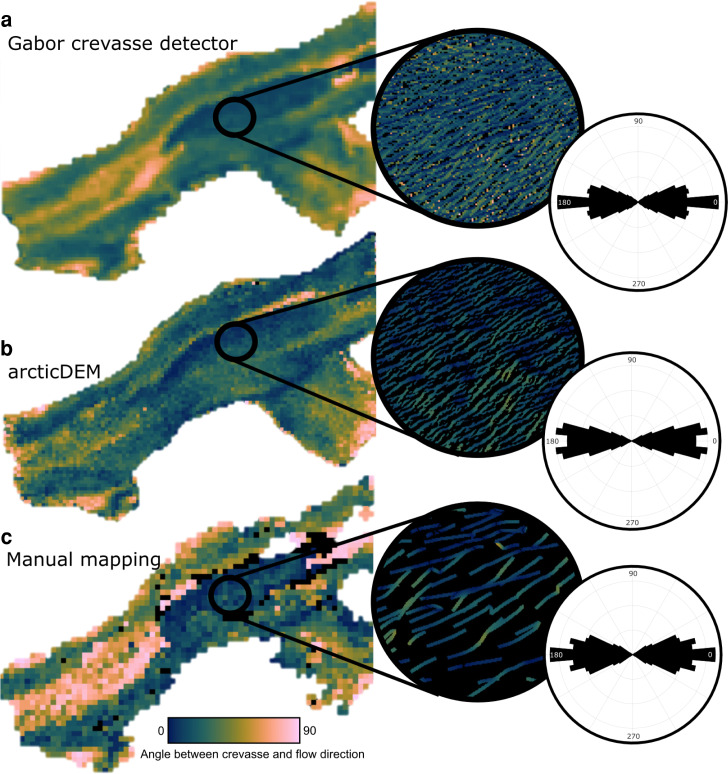
Comparison of measured crevasse orientation maps for the front of Narsap Sermia extracted using the GCD (a), DEM thresholding (b) and manual delineation (c). The inset shows the location and orientation of individual crevasses in one sub-region and a polar histogram of the angle between these crevasses and the direction of ice flow. All three methods identify a region of longitudinal crevassing 5–10 km up-glacier from the calving front.

## Supplementary material

For supplementary material accompanying this paper visit https://doi.org/10.1017/jog.2023.3. click here to view supplementary material

## Appendix A. The Gabor crevasse detector

We implement a method to automatically detect and calculate the orientation of crevasses within satellite images using a Gabor filter bank. This method is computationally rapid, extracts individual crevasse orientations and does not require a training dataset. A Gabor filter is a non-symmetrical 2-D Gaussian kernel, modulated in one direction by a sinusoid (Jain and Farrokhnia, 1990; Kruizinga and Petkov, 1999; Yang and others, 2015). A Gabor filter is therefore modulated by five main parameters: the orientation of the principal axes of the Gaussian *θ*, the standard deviation of the Gaussian in the rotated *x* and *y* directions, the wavenumber of the modulating sine wave *λ* and the phase of the sinusoid *ψ*. The complex Gabor filter *G*^*θ*^(*x*,  *y*) with a given angle *θ* is calculated:

A.1

in which *x*_*θ*_ and *y*_*θ*_ represent the original coordinate system rotated by a given angle *θ*:

A.2

and

A.3

Convolving a given Gabor filter *G*^*θ*^(*x*,  *y*) with a given image of a glacier *I*(*x*,  *y*) produces a Gabor response map :

A.4

With a complex Gabor kernel, the response map is also complex with a real and imaginary component. We calculate the final crevasse likelihood map *C*_L_ and crevasse orientation map *C*_*θ*_ by taking the maxima of the Gabor phase at each pixel:

A.5

with  and  being the real and imaginary parts of an individual Gabor response map respectively, and  representing the maximum across all individual Gabor response maps. The crevasse likelihood is calculated as the maximum value of all individual Gabor response maps, while the crevasse orientation is calculated as the Gabor filter angle *θ* which produces this maximum value. We then generate a binary crevasse mask *C*_B_ by thresholding the crevasse likelihood map, with pixels exceeding the empirically calibrated threshold of [1.25 median(*C*_L_)] classified as crevasses. Orientations of pixels below the crevasse classification threshold are also discarded. A fully commented implementation of the GCD is available with an example run at https://doi.org/10.5281/zenodo.7319274.

## Appendix B. Ice velocity calculation workflow

We calculate displacements from each Sentinel-2 image pair using iteratively reducing multipass template matching following the standard GIV workflow (Van Wyk de Vries and Wickert, 2021). We use the standard GIV reference window sizes of 1280, 640 and 320 m (128, 64 and 32 pixels) and a 75% window overlap, for a final velocity-map resolution of 80 m. Displacements are evaluated to sub-pixel precision in the final pass through a Taylor series expansion of correlation values neighbouring the integer extrema. Each pair-wise displacement map is converted into a velocity map by dividing it by the temporal separation between images. We then filter each velocity map by removing the value for pixels that have a signal-to-noise ratio lower than 5 or a peak ratio <1.3, exclude values that differ by more than 50% from their immediate neighbours (four surrounding cells) and 200% from the mean of their larger local area (25 surrounding cells), and interpolate across these now-empty pixels using the values of the remaining (i.e. valid) ice-speed pixels. We do not use a maximum velocity filter so as to capture rapid (>10 km a^−1^) motion of the glacier front and frontal melange. These thresholds were manually selected based on local tests to exclude the majority of pixels with erroneous velocity estimates based on comparison with neighborring pixels and reference to external datasets (Davison and others, 2020; Lei and others, 2021). To correct for georeferencing error, we fit a 2-D polynomial surface to the velocities of non-glacierized (stable) areas in the *x* and *y* directions for each image pair, and subtract this to the velocities across the entire image.

We calculate a time series of velocities for each pixel, and average all individual velocity maps into a median velocity map and flow direction map covering the entire 6 year period for each of the three image temporal separations (short, medium and long). We compare the velocity time series to the time series of measured crevasse density and orientation, while the median flow direction maps are used to calculate the angle between crevasses and ice flow direction. Nominal strain rates are calculated using the spatial gradient of the NS and EW components of velocity using a central difference scheme for interior data points and a single-sided difference scheme for the edge points. Our offset distance is double the resolution of our velocity grid except for edge points where it is equal to the grid resolution. A tensor rotation is then applied to these gradient maps according to the local flow direction to create the longitudinal strain, transverse strain and shear strain maps (Bindschadler and others, 1996; Alley and others, 2018).

## Appendix C. Evaluation of the GCD

We evaluate the GCD by comparing it to two other, previously used crevasse mapping techniques: DEM thresholding and manual delineation (Fig. 9). We obtain a high-resolution (2 mpixel^−1^) DEM of the front of Narsap Sermia from arcticDEM (Porter and others, 2018). Both the DEM and Sentinel-2 scene used to manually delineate crevasses were captured on the same day, 10/09/2016. We high-pass filter the DEM using a 50 × 50 pixel Gaussian kernel, and threshold the resulting elevation anomaly at −0.25 m to delineate crevasses. We manually delineate crevasses using a drawing tablet. We compare the distribution and orientation of crevasses obtained using the three methods. All three methods are correlated, with the binned crevasse orientations from the GCD having a Pearson's *r*-value of 0.51 and 0.53 with the DEM-derived and manually derived crevasse orientations. All three methods identify a pattern of primarily transverse crevasses in the 5 km nearest to the ice front, with a zone of longitudinal crevassing up-glacier of this. All three methods exhibit a similar histogram of relative crevasse-ice flow directions within the range of 0–25.
